# RNA Polymerase II Activity of Type 3 Pol III Promoters

**DOI:** 10.1016/j.omtn.2018.05.001

**Published:** 2018-05-08

**Authors:** Zongliang Gao, Elena Herrera-Carrillo, Ben Berkhout

**Affiliations:** 1 Laboratory of Experimental Virology, Department of Medical Microbiology, Academic Medical Center, University of Amsterdam, Amsterdam, the Netherlands

**Keywords:** type 3 Pol III promoters, Pol II, Pol III, dual-polymerase activity, promoter competition

## Abstract

In eukaryotes, three RNA polymerases (Pol I, II, and III) are responsible for the transcription of distinct subsets of genes. Gene-external type 3 Pol III promoters use defined transcription start and termination sites, and they are, therefore, widely used for small RNA expression, including short hairpin RNAs in RNAi applications and guide RNAs in CRISPR-Cas systems. We report that all three commonly used human Pol III promoters (7SK, U6, and H1) mediate luciferase reporter gene expression, which indicates Pol II activity, but to a different extent (H1 ≫ U6 > 7SK). We demonstrate that these promoters can recruit Pol II for transcribing extended messenger transcripts. Intriguingly, selective inhibition of Pol II stimulates the Pol III activity and vice versa, suggesting that two polymerase complexes compete for promoter usage. Pol II initiates transcription at the regular Pol III start site on the 7SK and U6 promoters, but Pol II transcription on the most active H1 promoter starts 8 nt upstream of the Pol III start site. This study provides functional evidence for the close relationship of Pol II and Pol III transcription. These mechanistic insights are important for optimal use of Pol III promoters, and they offer additional flexibility for biotechnology applications of these genetic elements.

## Introduction

Transcription in eukaryotes is conducted by three RNA polymerase (Pol) enzymes: Pol I, II, and III. These polymerases contain respectively 14, 12, and 17 subunits and share a 10-subunit catalytic core,^1^ but they synthesize distinct classes of cellular transcripts. Pol I transcribes large rRNAs; Pol II synthesizes mRNAs and most small nuclear RNAs (snRNAs); and Pol III uniquely transcribes small non-coding RNAs, including 5S rRNA (type 1), tRNAs (type 2), and other essential RNAs (type 3) such as the U6 snRNA.^2^

Type 3 promoters are unique among Pol III elements in that they solely utilize upstream regulatory elements, which resemble the architecture of canonical mRNA-type Pol II promoters.^3^ These gene-external Pol III promoters use a defined +1 transcription start site and recognize T stretches as a termination signal.^4^, ^5^, ^6^ Type 3 Pol III promoters, such as 7SK, U6, and H1, can, therefore, be used for the expression of almost any small RNA, including popular molecules like short hairpin RNA (shRNA) in RNAi applications and guide RNA (gRNA) in CRISPR-Cas9 genome-editing platforms.^7^, ^8^

Most knowledge of type 3 Pol III promoters is based on comprehensive studies of the U6 promoter that encodes the U6 snRNA.^9^, ^10^, ^11^ Among the five snRNAs required for pre-mRNA splicing, U6 snRNA is the only one that is transcribed by Pol III. The U6 promoter requires *cis*-acting elements that are similar to elements of Pol II promoters, including the proximal and distal sequence elements (PSE and DSE) of snRNA-type Pol II promoters and the TATA box of mRNA-type Pol II promoters.^4^, ^9^, ^10^, ^11^ A mutational analysis indicated that the TATA box is critical for Pol III activity.^11^ Interestingly, some Pol II activity type could be detected in this mutant U6 context,^11^ and similar results were described for the H1 promoter.^12^ Previous studies reported that Pol II binds near some Pol III genes, including 7SK-, U6-, and H1-transcribed genes.^13^ Selective inhibition of Pol II by α-amanitin was reported to reduce the expression of Pol III genes such as 7SK and U6.^14^, ^15^ It was proposed that Pol II may facilitate Pol III transcription by creating an active chromatin structure.^3^, ^14^ Pol II activity of these Pol III promoters is also suggested by their ability to drive Firefly luciferase (Luc) expression,^16^ but others suggested that Pol III is solely responsible.^17^ In general, Pol III transcripts are not suitable for protein translation because they are usually too short and an mRNA requires a 7-methylguanosine (m^7^G) cap at the 5′ end and a 3′ polyadenylated tail. It thus is doubtful whether Pol III-expressed transcripts can act as an efficient template for protein synthesis.^18^, ^19^, ^20^

We now report that all three commonly used human type 3 Pol III promoters (7SK, U6, and H1) can drive Luc translation. By systematic molecular analysis, we demonstrate that these Pol III promoters synthesize extended Luc mRNA transcripts by the recruitment of Pol II. Thus, these Pol III promoters have dual-polymerase activity. These three promoters exhibit similar Pol III activity, but they differ profoundly in Pol II strength (H1 ≫ U6 > 7SK). We demonstrate that the Pol III activity can be boosted by selective inhibition of the Pol II activity and vice versa, indicating Pol II-III competition for binding to the same promoter sequences.

## Results

### The Type 3 Pol III Promoters 7SK, U6, and H1 Exhibit Similar Pol III Activity

**Figure 1:**
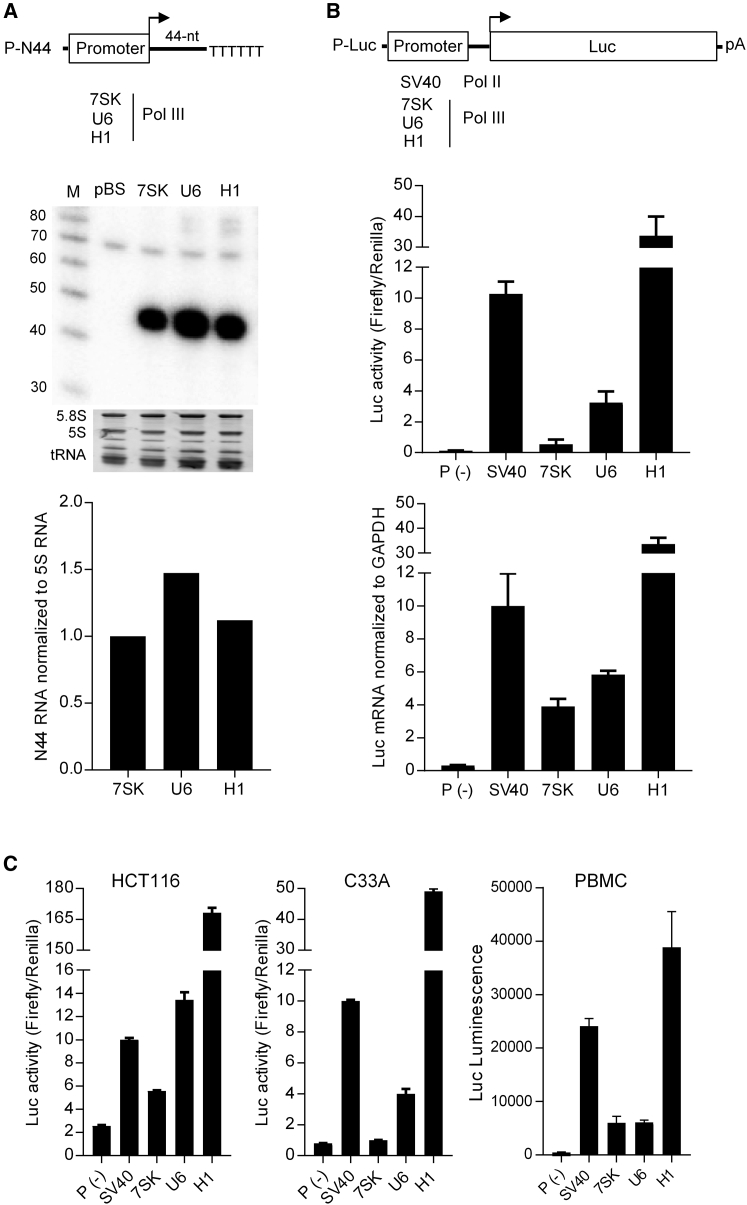
Small RNA and Luc Reporter Expression by Three Human Type 3 Pol III Promoters (7SK, U6, and H1) (A) Upper panel: P-N44 constructs encode Pol III promoters for transcription of an artificial 44-nt sequence, which terminates at the T6 signal. Middle panel: equimolar amounts of P-Luc constructs were transfected into HEK293T cells and total cellular RNA was harvested 36 hr post-transfection. The pBluescript (pBS) plasmid was used as a negative control. An equal amount of total RNA was subjected to northern blot for detection of the N44 RNA. M, RNA size marker (nt) is shown on the left. Ethidium bromide staining of rRNAs (5S and 5.8S) and tRNA serve as loading controls. Lower panel: quantitation of N44 RNA in middle panel. The N44 transcripts from the respective promoters were normalized to 5S RNA. 7SK-produced N44 was arbitrarily set at 1.0. The northern blot in (B) was repeated twice and very similar results were obtained. (B) Upper panel: design of the P-Luc constructs. Four promoters were inserted in the pGL3-basic backbone to drive Luc gene expression. The P(−) construct without any promoter was used as a negative control. Middle panel: Luc reporter activity of the respective promoters. Equimolar amounts of the P-Luc constructs and 1 ng Renilla plasmid to control for transfection efficiency were co-transfected into HEK293T cells. Dual-luciferase reporter assays were performed 36 hr after transfection, and the ratio of Firefly and Renilla luciferase was calculated to represent the Luc activity. The Luc activity measured for the SV40 promoter was arbitrarily set at 10. The results are presented as mean ± SD (n = 3). Lower panel: quantification of Luc mRNA made by different promoters. Total cellular RNA from P-Luc-transfected HEK293T cells was subjected to qPCR to quantitate the Luc mRNA level. The Luc RNA level for SV40 was arbitrarily set at 10. The GAPDH signal was used as an internal control. The data are shown as the mean ± SD (n = 3). (C) Luc expression in HCT116 and C33A cells and PBMCs. Luciferase assays for HCT116 and C33A cells were performed as in Figure 1B. The Luc activity measured for the SV40 promoter was arbitrarily set at 10. Equimolar amounts of P-Luc constructs were nucleotransfected into an equal number of PBMCs. After 24 hr, the firefly luciferase was measured and relative luminescence values were plotted.

To directly examine the Pol III activity of these human promoters, we constructed a set of P-N44 vectors in the same plasmid backbone in which the respective promoters transcribe an artificial 44-nt sequence (N44) followed by the efficient Pol III termination signal TTTTTT (T6) (Figure 1A, upper panel). Equal molar amounts of the three P-N44 constructs were transfected into HEK293T cells. Total cellular RNA was extracted 36 hr after transfection, and the same amount of total cellular RNA was subjected to northern blot analysis with a Pol47 probe that is complimentary to the N44 sequence. As expected, specific transcripts of ∼44-nt were detected corresponding to T6-mediated termination (Figure 1A, middle panel). The pBluescript (pBS) plasmid was used as a negative control. Quantification of the N44 RNA indicated similar Pol III activity for the three promoters, but U6 was a bit stronger than 7SK and H1 (Figure 1A, lower panel).

### Type 3 Pol III Promoters Drive Profoundly Different Levels of Luc Expression (H1 ≫ U6 > 7SK)

A previous report suggested cryptic Pol II mediated transcription from RNA Pol III promoters in shRNA expression vectors,^16^ but another study did not support this claim.^17^ We recently confirmed that the human H1 promoter can mediate Luc gene expression.^21^ To test if this is a common property of type 3 Pol III promoters, we used the set of three promoters to drive Luc expression. The standard SV40 early Pol II promoter was used as a positive control (Figure 1B, upper panel). Equal molar amounts of the otherwise isogenic P-Luc constructs were transfected into HEK293T cells, and a Renilla plasmid was co-transfected as a transfection control. Luciferase activity was measured after 36 hr, and the ratio of Firefly to Renilla was calculated to control for variation in the transfection efficiency. Significant Luc expression was induced by all four promoters compared with the promoter-less construct P(−) (Figure 1B, middle panel). Interestingly, the four promoters exhibited significant differences in strength. 7SK and U6 were ∼20- and ∼3-fold weaker than the SV40 promoter. Surprisingly, H1 seemed ∼3-fold stronger than the SV40 promoter. The results suggest that all three Pol III promoters can transcribe extended transcripts that are translation competent.

To directly measure the Luc mRNA levels, qPCR was carried out with equal amounts of total cellular RNA. The relative Luc mRNA level generally followed the variation observed in Luc activity (Figure 1B, lower panel). The combined results demonstrate that these three type 3 Pol III promoters can also produce extended transcripts that are translation competent. Two explanations can be envisioned to explain this phenomenon: Pol III readthrough or Pol II involvement.

To explore whether these Pol III promoters are able to induce Luc expression in other cell types, we tested these constructs in the HCT116 and C33A cell lines and primary peripheral blood mononuclear cells (PBMCs). The Luc expression trends were very similar in all cell types (Figure 1C). The 7SK promoter was the weakest and H1 the most potent promoter that was always stronger than the SV40 promoter. The results indicate that Luc expression from these Pol III promoters, especially the H1 promoter, is a general property that is observed in all cell types tested.

### Pol II Is Responsible for Luc Expression from Type 3 Pol III Promoters

**Figure 2:**
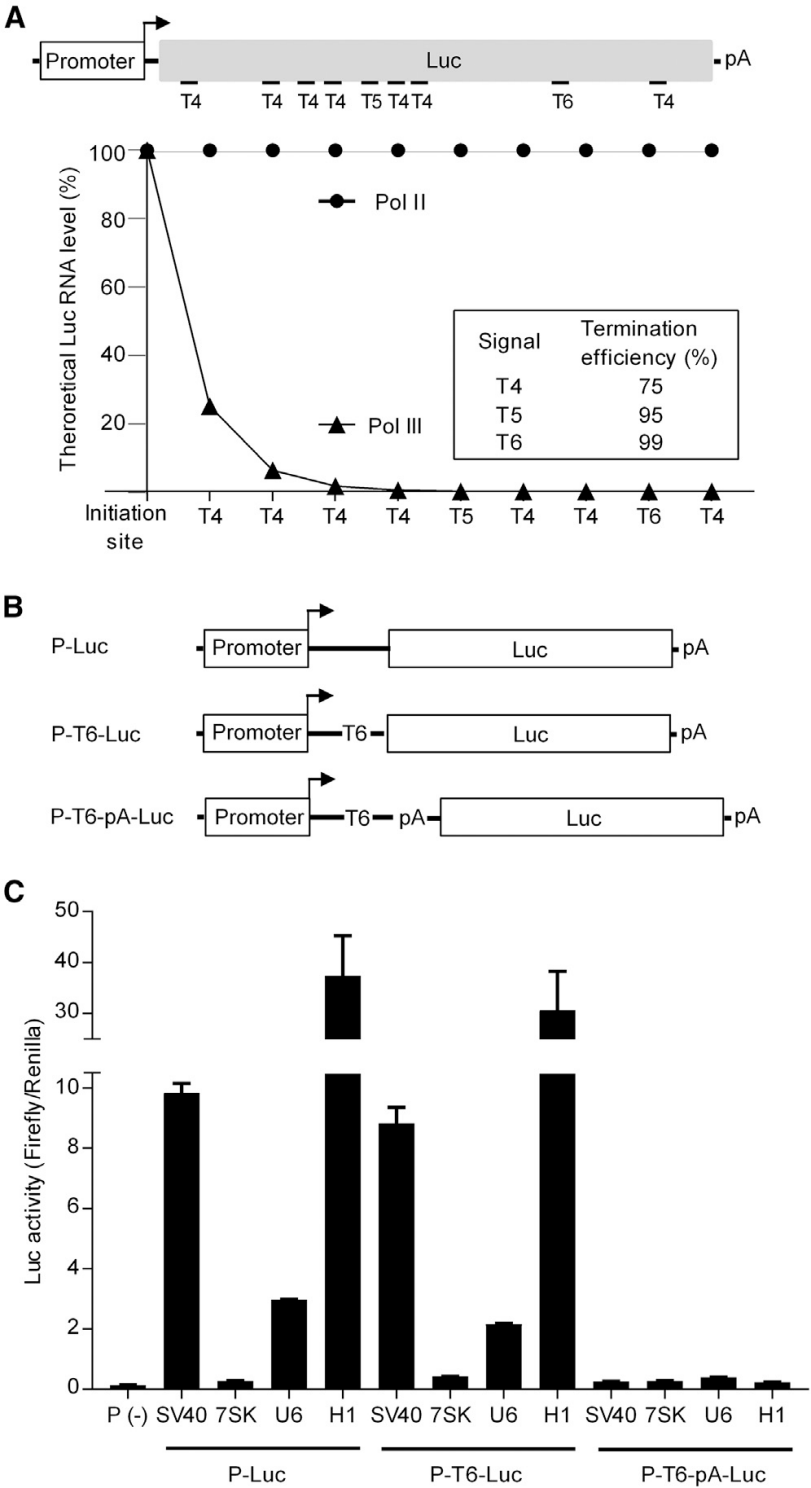
Pol III-Mediated Luc Expression: Sensitivity to Pol II/III Termination Signals (A) Theoretical full-length Luc mRNA level expressed by Pol II and III. Pol III termination signals (T_n_ stretch with n ≥ 4) in the Luc-coding sequence are indicated. Luc expression was predicted based on the Pol III termination efficiency by these T-stretch signals. (B) Luc constructs with added termination signals (T6 for Pol III and pA for Pol II). Signals were inserted between the promoter and Luc gene to create P-T6-Luc and P-T6-pA-Luc. (C) All three sets of constructs for 7SK, U6, and H1 promoters were transfected into HEK293T cells, and the Luc activity was determined as in Figure 1E.

To determine if Luc expression is due to Pol III readthrough transcription, we first inspected the Luc coding sequence for the presence of Pol III termination signals. This survey revealed 7 × T4, 1 × T5, and 1 × T6 signals (Figure 2A). We recently determined that the termination efficiency of U6 transcription is 75%, 95%, and 99% for a single T4, T5, and T6 signal, respectively.^22^ Accordingly, the theoretical drop-off of the Pol III transcript levels along the Luc gene was plotted (Figure 2A), which indicated that only a very minimal amount of Pol III transcripts (<0.001% of all initiated transcripts) were expected to read through all termination signals to produce a full-length Luc mRNA. In addition, Pol III transcripts would lack the 5′ cap and 3′ polyA tail that are needed for efficient translation. It is therefore very unlikely that Pol III is responsible for the observed Luc expression.

To confirm this idea, we inserted an efficient T6 Pol III termination signal upstream of the Luc gene in all four constructs (P-T6-Luc in Figure 2B). Transfection experiments and dual-luciferase reporter assays were performed as described above. The T6 signal had only a marginal inhibitory effect on Luc expression by the three Pol III promoters and, as expected, the SV40 Pol II promoter (Figure 2C). These results confirm that Pol III is not involved in Luc expression from the three Pol III promoters.

As a complementary approach, we inserted the SV40 late poly(A) signal (pA) upstream of the Luc gene (P-T6-pA-Luc in Figure 2B). As expected, this intervention profoundly knocked down Luc expression for the Pol II SV40 promoter, but the same was true for all three Pol III constructs (Figure 2C). All results argue that Pol II transcription occurs, with variable efficiency, on the three Pol III promoters.

**Figure 3:**
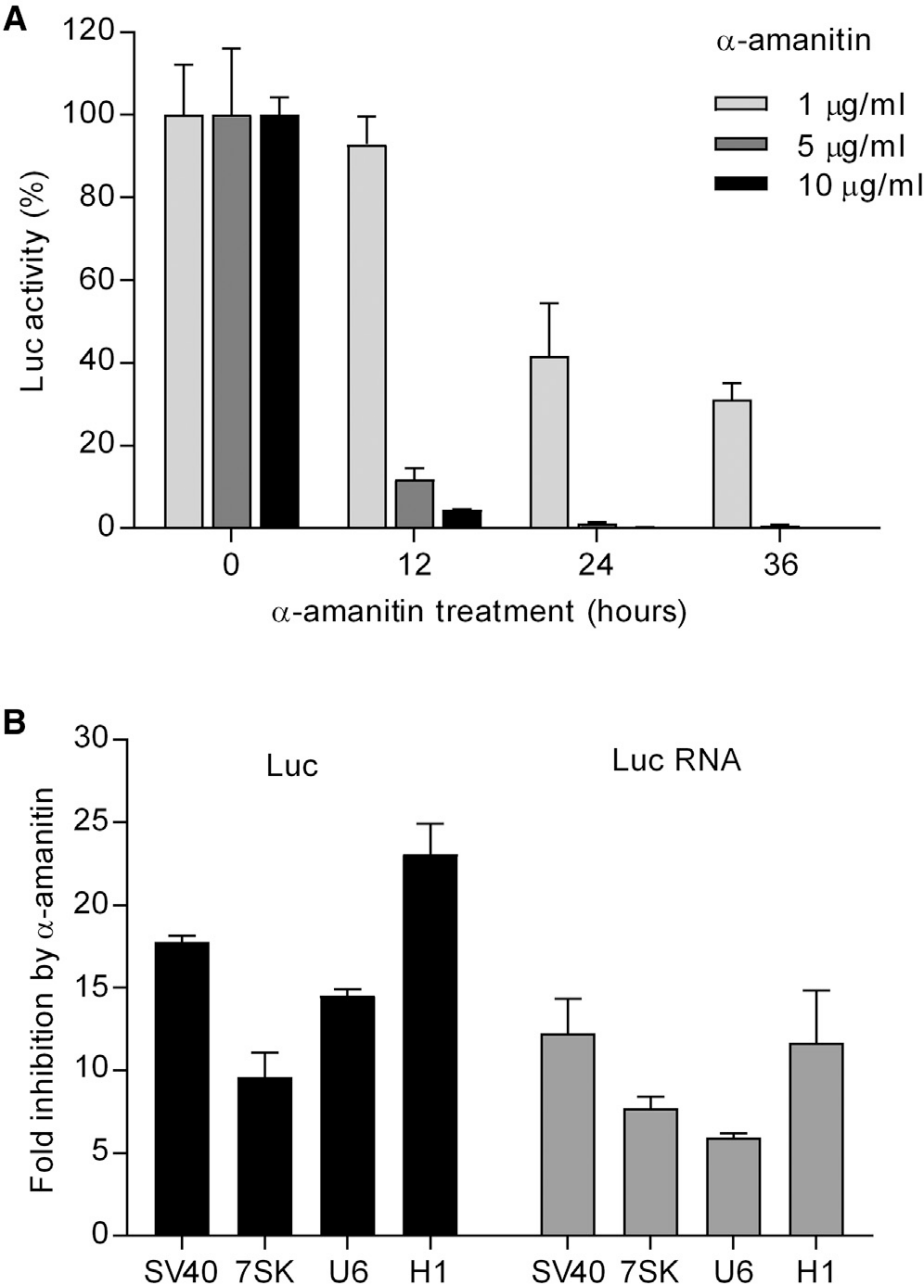
Luc Expression from Both Pol II and Pol III Promoters Is Sensitive to α-Amanitin (A) The influence of α-amanitin on SV40-mediated Luc expression. The P(SV40)-Luc construct was transfected into HEK293T cells, and cells were treated with α-amanitin for 0, 12, 24, or 36 hr at different concentrations (1, 5, or 10 μg/mL). The Luc activity was measured 36 hr after transfection. Luc activity without α-amanitin (0 hr of treatment) was arbitrarily set at 100%. (B) α-Amanitin significantly inhibits Luc activity and Luc RNA expression of Pol II/III promoters. The four P-Luc constructs were individually transfected with the pRL plasmid into HEK293T cells with or without 2 μg/mL α-amanitin. The dual-luciferase reporter assays were performed 36 hr post-transfection, and the fold inhibition of Luc activity by α-amanitin was calculated. Variation between constructs in the fold induction measured for the CMV promoter-driven Renilla construct (CMV-Renilla) was used for normalization. Total cellular RNA from the same experiments was subjected to qPCR, and GAPDH served as the internal control. The fold inhibition by α-amanitin is plotted and the data are presented as mean ± SD (n = 3).

As an independent test for Pol II involvement, we tested the impact of the Pol II-specific inhibitor α-amanitin, which binds to the catalytic domain to block transcriptional elongation.^23^ Pol I is not inhibited by α-amanitin, Pol II is highly sensitive, and Pol III is only mildly sensitive to α-amanitin. We first titrated α-amanitin against the Pol II SV40-Luc construct (Figure 3A). Transfected HEK293T cells were treated with different concentrations (1, 5, or 10 μg/mL) of α-amanitin for 0, 12, 24, or 36 hr, and Luc activity was determined 36 hr post-transfection. As expected, the SV40 Pol II promoter was highly sensitive to low concentrations of α-amanitin, and the greatest effect was scored by the early addition, likely because α-amanitin uptake by cells is a slow process. Similar results were obtained for the Pol II promoter of CMV (data not shown). For the Pol III promoter test, we chose 2 μg/mL α-amanitin, a concentration that is commonly used for selective Pol II inhibition in living cells.^24^ We plotted the fold inhibition of Luc expression (Figure 3B, left). All three Pol III constructs were sensitive to α-amanitin. H1 was most sensitive, even more than SV40, which correlates with the measured Pol II activity. A qPCR analysis confirmed that this effect took place at the Luc RNA level (Figure 3B, right). The combined data demonstrate that the 7SK, U6, and H1 promoters exhibit both Pol III and Pol II activity, the latter in the following ranking order: (H1 ≫ U6 > 7SK).

### Direct Visualization of Extended Pol II Transcripts Made from Pol III Promoters

**Figure 4:**
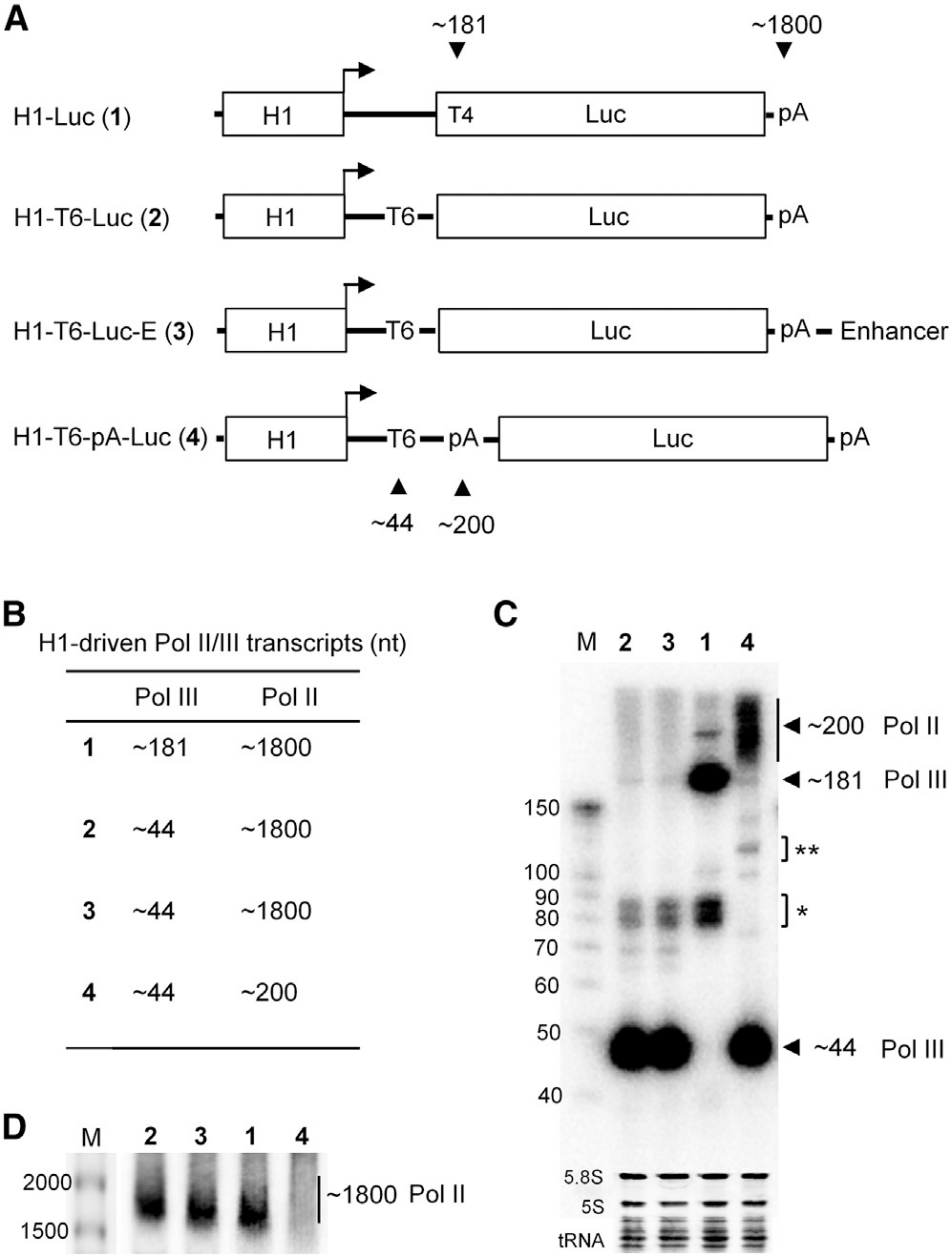
Simultaneous Visualization of Pol III and II Transcripts Made by the H1 Promoter (A) Four H1 promoter-based constructs with the termination signals (T6 for Pol III and pA for Pol II) are indicated. (B) Predicted transcript lengths. (C) Probing of Pol III/II transcripts on northern blot. Total cellular RNA from DNA-transfected HEK293T cells was isolated, and a fixed amount (5 μg) was subjected to northern blotting. The RNA size marker M was used for estimation of the transcript sizes. The rRNAs and tRNAs were stained with ethidium bromide and used as a loading control. The Pol III and Pol II transcripts are indicated (*minor Pol III transcripts of ∼80 nt that likely reflect termination at non-T6 signals; this becomes signal **for construct 4 due to the change in the local sequences). (D) Probing of ∼1,800-nt Pol II transcripts on northern blot. A fixed amount (15 μg) of total RNA was subjected to agarose-formaldehyde gel electrophoresis and northern blotting. The ethidium bromide staining of an RNA ladder was used for the estimation of transcript sizes.

Next, we wanted to directly compare the efficiency of Pol III versus Pol II activity on these Pol III promoters using northern blot detection. We first tested the H1 promoter that exhibits the most Pol II activity. Four H1-based constructs were transfected into HEK293T cells (Figure 4A). To faithfully evaluate both short Pol III and extended Pol II transcripts, equal amounts of total cellular RNA were subjected to northern blotting in combination with the Pol47 probe that detects both transcripts. The predicted sizes of Pol III and Pol II transcripts made by these four constructs are listed (Figure 4B). For the H1-T6-Luc (2) and H1-T6-Luc-E (3) constructs, a very similar transcription profile was apparent with a profound signal of ∼44 nt corresponding to Pol III termination at T6, which was absent for H1-Luc (1) (Figure 4C). This result indicates that the SV40 Pol II enhancer (E) in H1-T6-Luc-E (3) does not affect Pol III transcription, as could be expected. Transcripts terminated for H1-Luc (1) at the expected first T-stretch to create a Pol III transcript of ∼181 nt. No extended Pol II transcripts of ∼1,800 nt were detected for these three constructs on the 15% polyacrylamide gel that is ideally suited to separate transcripts up to 800 nt (Figure 4C).

To visualize the extended Pol II transcripts, we first set out to shorten the H1-generated Pol II transcripts by purposely moving the pA signal upstream of the Luc gene in H1-T6-pA-Luc (4) to generate ∼200-nt transcripts. Two major signals were observed: the ∼44-nt Pol III and the ∼200-nt Pol II signals (Figure 4C). The latter signal formed a smear of different length products, which is likely due to the post-transcriptional addition of a poly(A)-tail of variable length. Thus, the H1-T6-pA-Luc (4) construct provides a proper backbone for simultaneous assessment of Pol II and Pol III transcripts. Signal quantitation indicated approximately 3-fold more Pol III than Pol II transcription on the H1 promoter. Based on the differential Luc expression of the three Pol III promoters (Figure 2B), we cautiously estimated this ratio to increase to 50-fold and 500-fold for the U6 and 7SK promoters. We also wanted to detect the full-length ∼1,800-nt Pol II transcripts made by constructs 1–3. To do so, we performed agarose-formaldehyde gel electrophoresis and northern blotting. Transcripts of ∼1,800 nt were detected for constructs 1, 2, and 3, but not for construct 4 (Figure 4D), which correlates with expression of the ∼200-nt Pol II transcripts for construct 4 (Figure 4C).

### Pol III and Pol II Compete for Promoter Usage

**Figure 5:**
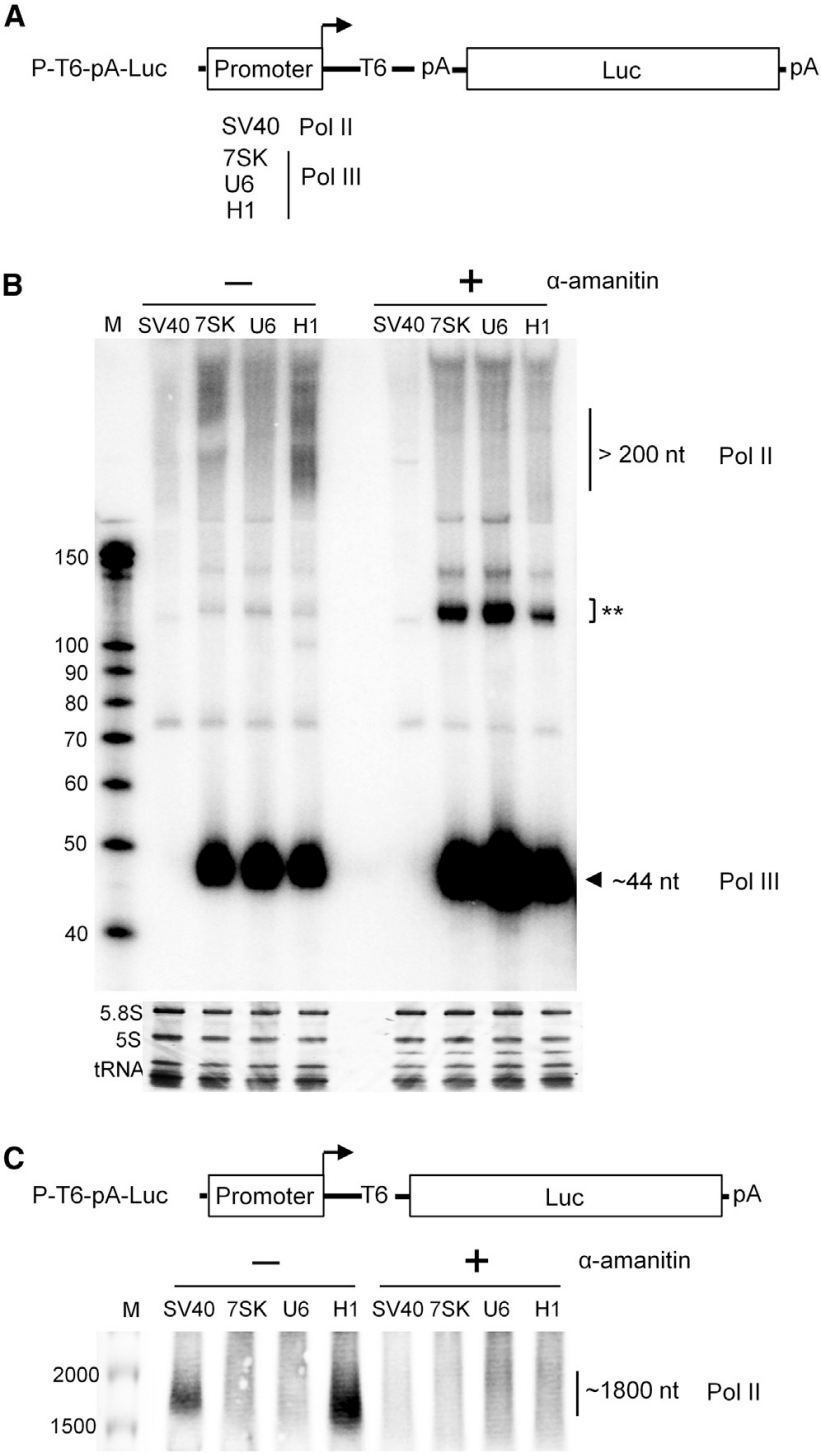
The Effect of α-Amanitin on Transcription from Pol III Promoters (A) Pol II/III promoters were cloned into the P-T6-pA-Luc backbone. (B) The P-T6-pA-Luc constructs were individually transfected into HEK293T cells with or without α-amanitin (2 μg/mL) treatment. RNA extraction and northern blotting were performed as in Figure 4C. The Pol III/II transcripts are indicated. The ** signals reflect minor Pol III transcripts (see legend to Figure 4C). The results were reproduced in a second independent experiment. (C) The P-T6-Luc constructs were individually transfected into HEK293T cells with or without α-amanitin (2 μg/mL). RNA extraction and northern blotting were performed as in Figure 4D.

We next attempted to apply the P-T6-pA-Luc design to all three Pol III promoters for the simultaneous visualization of Pol II and III transcripts (Figure 5A). The SV40 Pol II promoter was used as a positive control and the four constructs were transfected into HEK293T cells. An equal amount of harvested total cellular RNA was subjected to northern blot analysis. The ∼44-nt Pol III transcripts were observed at similar intensities, but larger variation was scored for the diffuse signals that represented ∼200-nt Pol II transcripts (Figure 5B, left). Consistent with the Luc data, the H1 promoter yielded the strongest Pol II signal. We also performed this test in the presence of 2 μg/mL α-amanitin (Figure 5B, right). Disappearance of the extended Pol II transcripts from SV40, 7SK, and H1 was most apparent. Intriguingly, the intensity of the ∼44-nt Pol III transcripts increased at least 2-fold by α-amanitin treatment. An additional signal of ∼120 nt was apparent (Figure 5B, right), which may represent Pol III readthrough transcripts. Thus, α-amanitin suppresses Pol II transcription and simultaneously increases Pol III activity, suggesting that the two polymerases compete for usage of the same promoter sequences.

We next tested the Luc mRNA transcription of the P-T6-Luc constructs with or without α-amanitin treatment. As in Figure 4D, samples were subjected to agarose-formaldehyde gel electrophoresis and northern blotting. A prominent ∼1,800-nt band corresponding to the Luc transcript was apparent only for the SV40 and H1 constructs (Figure 5C). The band intensity was consistent with the Pol II promoter strength as shown in Figure 1B (middle panel). The ∼1,800-nt transcript disappeared upon α-amanitin treatment, which confirmed that it was made by Pol II.

### Mutation of the TATA Box Abolishes Pol III Transcription, but It Increases Pol II Activity

**Figure 6:**
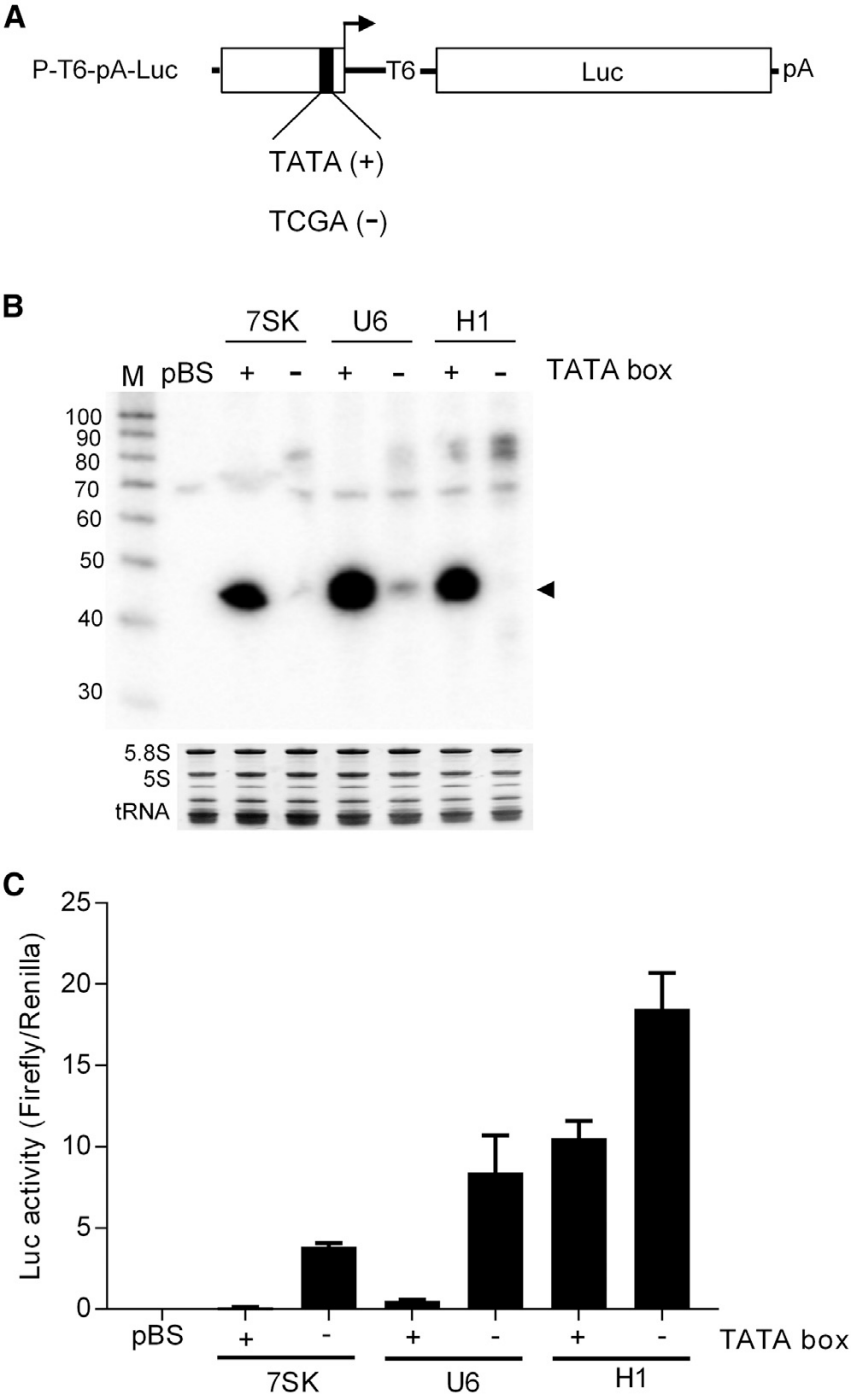
Inactivation of the TATA Box of Type 3 Pol III Promoters Abolishes Pol III Transcription, but It Stimulates Pol II Activity (A) Mutation of the TATA boxes of the three Pol III promoters was achieved by changing the TATA sequence (+) to TCGA (−). (B) Northern blot analysis was performed as described in Figure 4C. The pBS construct was used as a negative control. The ∼44-nt transcripts (black triangle) represent Pol III transcripts terminated at T6. (C) The Luc activity of the different promoter constructs with (+) or without (−) TATA box. The pBS served as a negative control. The data are shown as mean values ± SD (n = 3).

The TATA box of Pol III promoters acts as a major determinant of Pol III specificity.^25^ We mutated the TATA box in the three P-T6-Luc constructs (Figure 6A). As depicted in Figure 6B, this mutation abolished Pol III activity, consistent with previous reports.^5^, ^11^, ^12^ Intriguingly, Pol II activity as measured by Luc expression was significantly enhanced for all three promoters by mutation of the TATA box (Figure 6C). This result supports the idea of Pol II-III competition at these promoters.

### Mapping the Pol II Transcription Start Site on Pol III Promoters

**Figure 7:**
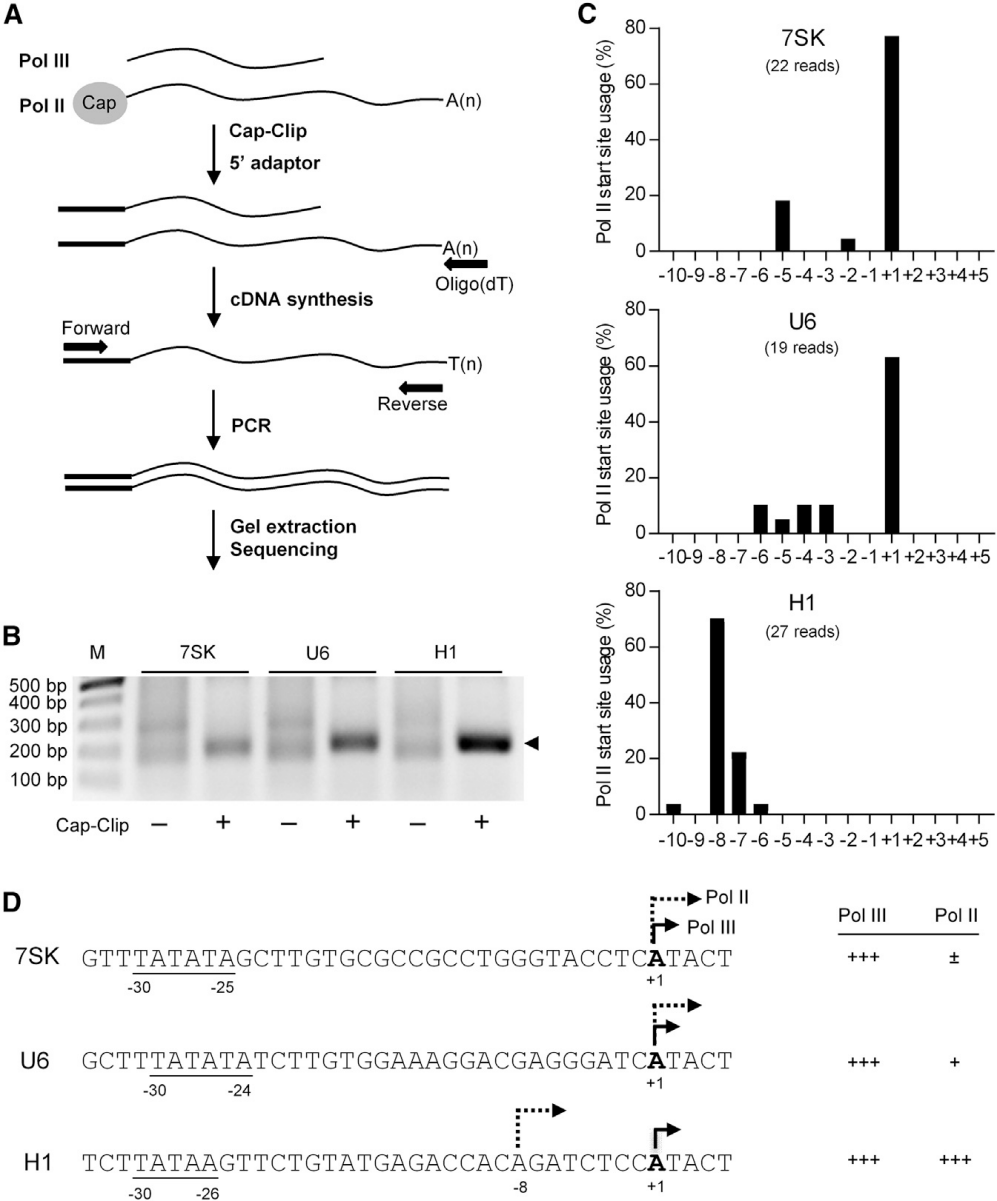
Mapping the Pol II Transcription Start Site on Type 3 Pol III Promoters (A) Schematic of the mRNA 5′-RACE procedure. The Cap-Clip Acid Pyrophosphatase is used to specifically hydrolyze the 5′ cap of mRNA. (B) Amplification of Pol II-specific transcripts made by the three promoters. The 5′-RACE was performed with (+) or without (−) Cap-Clip treatment. Products of the expected size are marked by a black triangle. (C) Illustration of Pol II start site usage on Pol III promoters. The Cap-Clip-dependent products from (B) were subjected to TA-cloning and Sanger sequencing. The Pol II transcription start site was determined by aligning the sequencing output with the DNA construct. The start position was related to the position of Pol III transcription initiation, which was arbitrarily defined as +1. (D) Sequences around the Pol III/II transcription start sites. Pol III start sites (solid arrow) and Pol II start sites (dotted arrow) are indicated. The TATA boxes of these Pol III promoters are underlined and the position is indicated. The relative Pol III and II strengths (right panel) were derived from the results represented in Figure 1.

Finally, we set out to determine where Pol II transcription initiates on these Pol III promoters. We used the 5′-rapid amplification of cDNA ends (RACE) method to map the 5′ end of the Luc mRNA, which reflects the transcriptional start site in the promoter DNA. An equal amount of total cellular RNA from P-Luc-transfected HEK293T cells was used as a template. To specifically amplify Pol II transcripts, 5′ cap-removing reagent (Cap-Clip) and the Oligo(dT) primer were used for cDNA synthesis (Figure 7A). As shown in Figure 7B, Cap-Clip-specific bands were amplified for all three constructs. The dependence on Cap-removing reagent indicated that the Pol II transcripts made by these Pol III promoters looked like regular mRNAs, which are 5′-capped and 3′-polyadenylated. The intensity of these signals correlated with the measured Pol II strength (H1 ≫ U6 > 7SK). We mapped the transcription start sites by sequencing of the Pol II-specific PCR products (Figure 7C). Pol II transcription from the 7SK and U6 promoters started predominantly at the well-known Pol III start site (Figure 7D). However, the H1 promoter used a novel Pol II start site around 8 nt upstream of the Pol III +1 start site.

## Discussion

Type 3 Pol III promoters have been widely used for synthesis of small functional RNAs, such as the shRNA in RNAi applications and gRNA in CRISPR-Cas genome-editing platforms. In this study, we report that three commonly used human Pol III promoters (7SK, U6, and H1) also have Pol II activity, with significant variation in strength (H1 ≫ U6 > 7SK). Pol II activity of the H1 promoter reaches approximately 30% of the Pol III activity, and this Pol II activity surpasses that of the regular early SV40 Pol II promoter. These Pol III promoters can produce extended mRNAs that are translation competent. The selective inhibition of either polymerase (II with α-amanitin or III by TATA-box mutation) stimulates promoter usage by the other polymerase, indicating that they compete for binding to the promoter.

Extensive transcription studies of the human Pol II snRNA and Pol III U6 snRNA promoters revealed close similarity in their structure.^25^ Both promoters require the PSE for basal transcription and the DSE to enhance transcription. These elements are interchangeable between these Pol II snRNA and Pol III U6 promoters.^11^, ^25^ The U6 Pol III activity requires an additional TATA box,^11^, ^25^, ^26^ but other elements are likely to favor Pol II recruitment for snRNA transcription, but not mRNA expression.^27^, ^28^, ^29^, ^30^ In contrast, a Pol II snRNA promoter was reported to express protein-coding genes.^31^, ^32^ Combined with the data on Luc expression from Pol III promoters described in this study, it can be concluded that snRNA promoters can express mRNA for protein production.

The tested Pol III promoters show significant variation in Pol II strength (H1 ≫ U6 > 7SK). Pol II on the 7SK and U6 promoters initiates from the regular Pol III +1 site, whereas Pol II on H1 starts 8 nt upstream of the regular Pol III +1 position. These striking differences are likely due to a variation in promoter structure. The 7SK and U6 promoters have a very similar architecture with the DSE/PSE motifs at a distance of ∼150 bp,^25^ but the DSE is adjacent to the PSE in the unusually compact H1 promoter.^12^ This particular H1 promoter architecture may favor Pol II recruitment and induce the shift in Pol II start site. It will be of interest to study the physiological role of Pol II transcription from the cellular H1 promoter, e.g., by checking if a protein-coding mRNA is generated.

There is accumulating evidence for an important regulatory role of chromatin structure on Pol III promoter activity.^33^ Genome-wide analysis of the Pol III transcriptome in HeLa cells revealed significant overlap of Pol III genes with active chromatin.^3^ In addition, the chromatin-binding protein CHD8 was reported not only to contribute to Pol II-mediated mRNA transcription but also to promote U6 Pol III transcription.^34^ Selective inhibition of Pol II by α-amanitin reduced the expression of Pol III genes, including 7SK and U6, suggesting that Pol II may influence Pol III transcription on chromatin DNA.^14^, ^15^ Furthermore, chromatin immunoprecipitation sequencing (ChIP-seq) experiments revealed that Pol II binds near Pol III genes.^14^ Pol II co-occupancy was reported in many Pol III genes, including 7SK-, U6-, and H1-transcribed genes.^3^, ^13^ Pol II-III co-occupancy was most prominent for the H1 gene, which coincides with the highest Pol II activity scored in this study. It was proposed that Pol III transcription relies on initial Pol II binding to recruit transcription factors that create an active chromatin structure.^3^, ^14^ For instance, the initiation factor PSE-binding factor (PTF) is required to maintain an open chromatin structure across the human snRNA genes for efficient transcription.^35^ This action may occur through interaction with chromatin remodelers like CHD8, which is recruited to the U6 promoter.^34^ In this study with chromatin-free plasmid templates, we show that the selective inhibition of Pol II enhances Pol III activity. We like to present the following consensus on these seemingly contradictory findings: on plasmid DNA, there is direct competition between the two polymerases, but on chromatin DNA, the recruited Pol II is required to induce an accessible chromatin structure that facilitates Pol III transcription.

The dual-polymerase activity of these type 3 Pol III promoters has important implications for small RNA expression. A few potential adverse effects of unwanted Pol II transcripts can be predicted. For therapeutic H1-shRNA vectors, the extended Pol II transcripts will have the perfect complementary target sequence and may be attacked by the Pol III-generated shRNA, and this may comprise the therapeutic silencing efficiency. The strong Pol II activity of H1 promoter may also interfere with the activity of downstream Pol II transcription units.^36^ Of the three promoters investigated in this study, 7SK seems the best choice for pure small RNA expression. Noticeably, the most frequently used U6 promoter exhibits some Pol II activity, which may cause unwanted side effects. Surprisingly, the H1 promoter exhibits robust Pol II activity and, therefore, seems less suitable for small RNA expression.

The dual-polymerase activity of H1 and U6 promoters may also be exploited for specific purposes. First, one could try to identify promoter mutants that have selectively lost the Pol II activity, thus constituting a pure Pol III promoter. Second, these promoters can be used as pure and strong Pol II promoter for protein synthesis by deleting Pol III-specific elements. As a proof of principle, we achieved this goal by mutation of the TATA box that kills Pol III transcription and, consequently, boosts Pol II-mediated Luc expression. Third, we envisage that the dual II-III activity could be used for the simultaneous expression of a small RNA and a protein. For example, the popular CRISPR-Cas9 genome-editing system requires a small gRNA plus the Cas9 protein, and both components could in theory be expressed from a single Pol III promoter. This would minimize the size and the complexity of transgene cassettes and benefit the construction of viral vectors with limited packaging capacity, such as adeno-associated virus and lentiviral vectors.^37^, ^38^ Future work should focus on the identification of Pol II/III-specific sequence elements that will allow us to fine-tune the Pol II/III ratio and to design promoters with exclusive II or III activity. Such reagents will provide more flexibility for specific biotechnology applications.

## Materials and Methods

### Construction of Vectors

The vectors psiRNA-h7SK hygro G1 (InvivoGen), pSilencer 2.0-U6 (Ambion), and pSUPER (Oligoengine) were used as sources of human Pol III promoter sequences. The U6 variant used was U6-1, which is one of the strongest U6 promoters.^39^ The N44 sequence (ACCATGGAAGTGAAGGGGCAGTAGTAATATACCGGTGATATCAC) followed by a T6 stretch was inserted downstream of the Pol III promoters in these vectors. The promoter-N44 fragments were amplified using specific primers that encode the same restriction enzyme sites (NheI and HindIII), digested, and ligated into the similarly opened pGL3-basic vector. Mutation of the T6 termination signal to TGTATT was achieved by a specific primer. The SV40 late pA signal was PCR amplified from pGL3-basic. The resulting PCR product was digested with HindIII and NcoI and inserted into the P-T6-Luc vectors. The DNA sequences of the three Pol III promoters with TATA box mutations were synthesized by Integrated DNA Technologies (IDT) and cloned into appropriate vectors by Gibson cloning according to the protocol (New England Biolabs). All constructs were verified by sequencing using the BigDye Terminator v1.1 Cycle Sequencing Kit (ABI).

### Cell Culture

HEK293T cells, C33A cells, and HCT116 cells were grown as monolayer in DMEM (Life Technologies) supplemented with 10% fetal calf serum (FCS), minimal essential medium nonessential amino acids, penicillin (100 U/mL), and streptomycin (100 μg/mL) at 37°C and 5% CO_2_. C33A is a human cervical cancer cell line and HCT116 is a human colon cancer cell line. Cells were trypsinized and seeded 1 day prior to transfection. At 1 day prior to transfection, 3 × 10^5^ and 1.5 × 10^6^ cells were seeded per well in 12-well plates and 25 cm^2^ flasks, respectively.

### Transfection of PBMCs by Means of Nucleofection

At 3 days before transfection, PBMCs were thawed and cultured in the presence of phytohemagglutinin (PHA, 2 μg/mL). Culture medium was supplemented with interleukin-2 (IL-2) to 1 μg/mL 1 day prior to transfection. 2 × 10^6^ PBMCs were transfected with 30 μg Luc constructs using the P3 Primary Cell 4D-Nucleofector X Kit (Lonza) with the EO-115 program. Luc expression was measured 24 hr post-transfection.

### Dual-Luciferase Reporter Assay

Equimolar amounts (equivalent of 200 ng empty pGL3-basic plasmid) of P-Luc constructs and 2 ng Renilla luciferase plasmid (pRL) were co-transfected into HEK293T cells in 12-well plates using Lipofectamine 2000 (Invitrogen), according to the manufacturer’s instructions. At 36 hr post-transfection, luciferase activity was measured with the dual-luciferase reporter assay system (Promega, Madison, WI, USA), according to the manufacturer’s protocol. The ratio of Firefly to Renilla was calculated to represent the relative Luc activity. Three independent transfections were performed. The luciferase data were corrected for between-session variation as described previously.^40^

### Treatment with α-Amanitin

In the initial test, different concentrations (1, 5, and 10 μg/mL) of α-amanitin (Sigma) were realized at different time points after transfection. 2 μg/mL α-amanitin was used in the dual-luciferase reporter and RNA extraction assays.

### Northern Blot Analysis

Northern blot for small RNAs detection was performed as previously described.^41^ Briefly, equimolar amounts (equivalent of 5 μg empty pGL3-basic plasmid) of P-N44 constructs were transfected into HEK293T cells in T25 flasks using Lipofectamine 2000 (Invitrogen). Total cellular RNA was extracted 36 hr post-transfection using the mirVana miRNA isolation kit (Ambion). Of total RNA, 5 μg was heated for 5 min at 95°C and then resolved in a 15% denaturing polyacrylamide gel (Precast Novex TBU gel, Life Technologies). The γ[^32^P]-labeled decade RNA marker (Life Technologies) was used for size estimation. To check for equal sample loading, the gel was stained in 2 μg/mL ethidium bromide for 20 min and visualized under UV light. The RNA in the gel was transferred to a positively charged nylon membrane (Boehringer Mannheim). Locked nucleic acid (LNA) oligonucleotides (Pol47: 5′-ATTACTACTGCCCCTTCAC-3′) were 5′ end labeled with the kinaseMax kit (Ambion) in the presence of 1 μL γ[^32^P]-ATP (0.37 MBq/μL, PerkinElmer). Sephadex G-25 spin columns (Amersham Biosciences) were used to remove the unincorporated nucleotides. The membrane was incubated overnight with labeled LNA oligonucleotides in 10 mL ULTRAhyb hybridization buffer at 42°C. The membrane was washed with low (2 × saline sodium citrate [SSC] and 0.1% SDS) and high (0.1 × SSC and 0.1% SDS) stringency buffers. The signals were captured by Typhoon FLA 9500 (GE Healthcare Life Sciences) and quantitated using ImageJ software.

For detection of extended RNA transcripts, agarose-formaldehyde gel electrophoresis and northern blotting were performed. 15 μg total cellular RNA was mixed with denaturing loading dye and heated for 10 min at 70°C, and then it was subjected to electrophoresis in 1% denaturing formaldehyde gels in 3-(N-morpholino)propanesulfonic acid (MOPS) buffer at 120 V for 4 hr. The RiboRuler High Range RNA Ladder (Thermo Fisher Scientific) was run alongside for size estimation. The RNA was transferred onto a positively charged nylon membrane using 20 × SSC (3.0 M NaCl and 0.3 M Na-citrate [pH 7.0]) overnight by capillary force. The RNA was cross-linked to the membrane by a UV crosslinker. The membrane was incubated for 1 hr in ULTRAhyb hybridization buffer at 55°C. The probe, consisting of the NcoI- and XbaI-digested luciferase fragment of pGL3-control, was denatured for 10 min at 96°C and labeled with [α-32P] dATP by using the DecaLabel DNA Labeling Kit (Thermo Fisher Scientific). The probe was purified on a Sephadex G-25 spin column and added to the prehybridized membrane and hybridized overnight at 55°C. The membrane was washed three times for 15 min at room temperature in low-stringency buffer and at 50°C in high-stringency buffer. Signals were obtained and analyzed as described above.

### Real-Time qPCR

Total cellular RNA was harvested 36 hr post-transfection using the mirVana miRNA isolation kit (Ambion). RNA purity (A_260_/A_280_ ratio ≥ 1.8) and concentration were measured using NanoDrop 2000. DNA-free RNA was obtained by RURBO DNase (Ambion) treatment to remove residual genomic DNA, according to the manufacturer’s instructions. Briefly, 5 μg total cellular RNA was resolved in 50 μL TURBO DNase Buffer and incubated with 1 μL TURBO DNase (2 U) at 37°C for 30 min. RNA was extracted with phenol/chloroform and dissolved in 9 μL nuclease-free water. The DNA-free RNA sample was reverse transcribed in a 20-μL reaction by the Oligo(dT) primer using the First-Strand cDNA Synthesis Kit (ThermoScript RT-PCR system). The qPCR reactions were prepared with SensiFAST SYBR No-ROX Kit (Bioline), according to the manufacturer’s instructions. Reactions were run on the LightCycler 480 (Roche) according to the amplification protocol: 5 min 95°C followed by 35 cycles of 10 s 95°C, 30 s 60°C, and 30 s 72°C. Primers for Luc and glyceraldehyde 3-phosphate dehydrogenase (GAPDH) were designed to amplify a fragment of about 150 bp in length and the primer sequences were as follows: Luc: 5′-GAGGCGAACTGTGTGTGAGA-3′, 5′-GTGTTCGTCTTCGTCCCAGT-3′; and GAPDH: 5′-ACAGTCAGCCGCATCTTCTT-3′, 5′-GAGTCAACGGATTTGGTCGT-3′. The Luc primers were designed to amplify a Luc fragment between positions 1,159 and 1,293. The raw data were exported and analyzed using *LinRegPCR* program, and the relative expression of Luc RNA was normalized to GAPDH.

### Mapping of the Pol II Transcription Start Site

Of total cellular RNA of P-Luc-transfected HEK293T cells, 10 μg was treated with RURBO DNase (Ambion) as described above. The DNA-free RNA was treated by Cap-Clip acid pyrophosphatase (CellScripts) to remove the 5′ cap from the mRNA molecules. Phenol-chloroform precipitation was used to remove the Cap-Clip enzyme. The resulting RNA was ligated to a 5′ RNA-adaptor (CGACUGGAGCACGAGGACACUGACAUGGACUGAAGGAGUAGAAA). The Oligo(dT) primer was used for reverse transcription using the First-Strand cDNA Synthesis Kit (ThermoScript RT-PCR system). PCR amplification was carried out using a 5′ adaptor forward primer (5′-AGGACACTGACATGGACTGAA-3′) and a gene-specific reverse primer (5′-CGGACATTTCGAAGTACTCA-3′). PCR products of the expected size were gel extracted and cloned into the pCR2.1 TOPO vector by TA cloning (Invitrogen). Colony PCR was performed with T7 and M13R primers. The amplified inserts were subjected to sequencing using the BigDye Terminator v1.1 Cycle Sequencing Kit (ABI). The Pol II transcription start site was determined by aligning the sequencing reads with the sequence in the corresponding P-Luc constructs.

## Author Contributions

Z.G., E.H.C., and B.B. designed the experiments. Z.G. and B.B. drafted the manuscript. Z.G. conducted the experiments, and all authors analyzed the data.

## Conflicts of Interest

The authors declare no conflicts of interest.
